# CDK4/6 and MAPK—Crosstalk as Opportunity for Cancer Treatment

**DOI:** 10.3390/ph13120418

**Published:** 2020-11-24

**Authors:** Lisa Scheiblecker, Karoline Kollmann, Veronika Sexl

**Affiliations:** Institute of Pharmacology and Toxicology, University of Veterinary Medicine Vienna, 1210 Vienna, Austria; lisa.scheiblecker@vetmeduni.ac.at (L.S.); karoline.kollmann@vetmeduni.ac.at (K.K.)

**Keywords:** CDK6, MAPK, p38, combinational, palbociclib, inhibitors, cancer

## Abstract

Despite the development of targeted therapies and novel inhibitors, cancer remains an undefeated disease. Resistance mechanisms arise quickly and alternative treatment options are urgently required, which may be partially met by drug combinations. Protein kinases as signaling switchboards are frequently deregulated in cancer and signify vulnerable nodes and potential therapeutic targets. We here focus on the cell cycle kinase CDK6 and on the MAPK pathway and on their interplay. We also provide an overview on clinical studies examining the effects of combinational treatments currently explored for several cancer types.

## 1. Introduction

**Figure 1:**
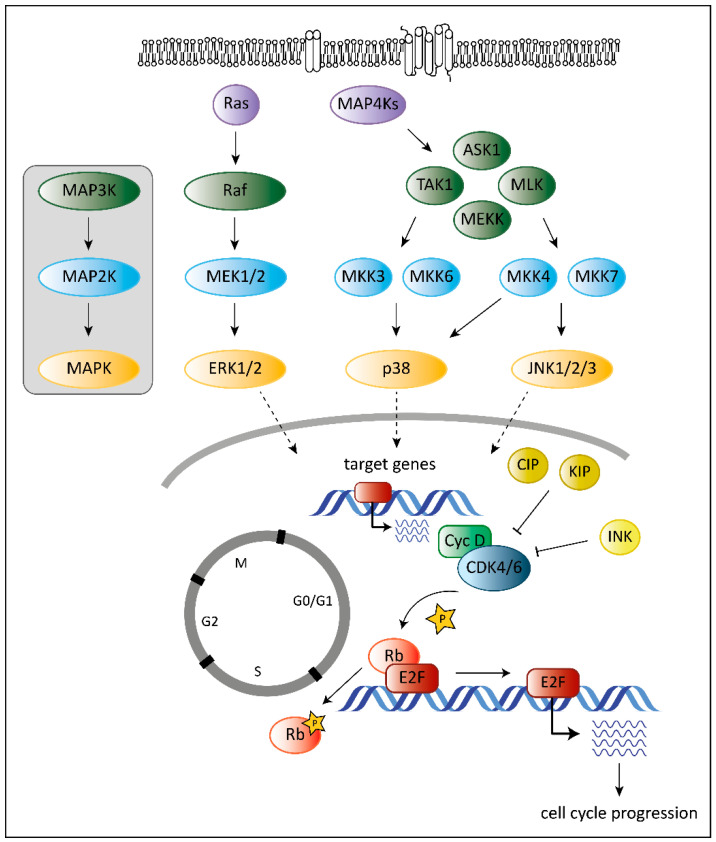
Overview of the best-studied MAPK signaling cascades and their interplay with the cell cycle machinery. MAPK cascades consist of three core kinases. The general pattern of the core kinases is shown in the box on the left. The three core kinases are activated by upstream kinases (MAP4Ks, Ras). Activation is indicated by the arrows. Activated MAPKs translocate into the nucleus (indicated by the dashed arrows) and induce expression of their target genes. Target genes of MAPKs are among others genes encoding D-type cyclins. They bind and activate cell cycle kinases CDK4 and 6 leading to phosphorylation on Rb (indicated by the star symbol) and to release of Rb-mediated repression of E2F target genes resulting in progression of the cell cycle to the S-phase. Members of the CIP/KIP and the INK protein families inhibit CDK4/6 as indicated by the “T-lines”.

Tumorigenesis requires that cells hijack regulatory networks to obtain their full oncogenic potential. The MAPK (mitogen-activated protein kinase) pathway is a tightly regulated signaling cascade frequently mutated or deregulated in cancer. Extra- and intracellular stimuli activate the MAPK cascade that regulates a broad variety of cellular programs including proliferation, differentiation, stress responses and apoptosis [1]. The best-studied MAPK pathways are the extracellular signal-regulated kinase (ERK) and stress-activated MAPK (c-Jun N-terminal kinase JNK and p38) pathways (Figure 1). While the ERK pathway is predominantly activated by growth factors to drive survival and cell growth, activation of the JNK or p38 pathway is accomplished by environmental stressors and inflammatory cytokines and is associated with apoptosis and growth inhibition [1]. MAPK cascades are structured in three core protein kinases (MAP3K, MAP2K and MAPK), which allows a tight regulation [1] (Figure 1).

The MAPK pathway interferes with the cell cycle machinery at many stages [1,2]. Transcriptional regulation of cell cycle components by MAPKs links cyclin-dependent kinases (CDKs) to the MAP kinase cascade. MAPKs regulate the expression levels of D-type cyclins, key players of the cell cycle [2]. While ERK signaling stimulates cyclin D1 expression upon growth factor exposure, p38 signaling suppresses cyclin D1 expression [2]. Changing the levels of D-type cyclins impacts on the activation of their cell cycle partners, the cyclin-dependent kinases 4 and 6 (CDK4 and CDK6). CDK4 and its close homologue CDK6 play a crucial role in various cancer types. Both are part of the G1 cell cycle checkpoint and allow the cell to enter into the S-phase [3]. When bound to D-type cyclins, CDK4/6 phosphorylate the retinoblastoma (Rb) proteins to release Rb-mediated repression on E2F target genes that drive G1 to S progression [4,5] (Figure 1).

Another layer of complexity is added as the ERK pathway regulates cell cycle progression dependent on the intensity and duration of the stimulating signal [6,7]. Whereas pronounced Raf activation triggers the induction of CDK4/6 inhibitor p21^Cip1^ and inhibits G1/S progression, moderate Raf signaling promotes DNA synthesis and expression of cyclin D1 [7].

In addition, p38 stabilizes the p21^Cip1^ protein [8]. High p21^Cip1^ levels induce a G1 arrest by binding to and inactivating G1 phase CDKs [9]. However, p38 also affects later phases of the cell cycle by interfering at the G2/M checkpoint. p38 activation initiates a G2 arrest as the immediate response to UV exposure [10,11]. Thus p38 serves as a sensor of DNA damage and puts the break on the cell cycle at the G2/M border.

The MAPK cascade and the CDK4/6 signaling complex are frequently deregulated or mutated in a variety of cancer types, which prompted the development of small molecular weight inhibitors that made it to clinic. Their impact is limited by resistance development, which evolves rapidly when drugs are used as monotherapy. One important strategy to avoid resistance development is the use of drug combinations. On the one hand combinational treatment may lead to a more efficient eradication of tumor cells if the drugs act synergistically. On the other hand it may support resensitization of tumor cells with acquired resistance. Tumors treated with MAPK inhibitors often acquire resistance after only a short period. One prominent mechanism of acquired resistance in this context is reactivation of the MAPK pathway and upregulation of cyclin D1 [12]. Simultaneous inhibition of CDK4/6, the main binding partner of cyclin D thus represents a promising treatment strategy to block proliferation of tumor cells and delay or overcome resistance to MAPK inhibitors. 

We here discuss the interference of CDK6 and MAPK cascades in cancer and focus on currently available inhibitors in preclinical and clinical contexts, which may open therapeutic options for combined targeted therapy.

## 2. The Role of CDK6 in Cancer

Cyclin D–CDK4/6 activation is frequently deregulated in cancer. Despite overlapping functions in cell cycle regulation, CDK4 and CDK6 dysregulation is linked to different cancer types. High levels of CDK4 are common in sarcoma [13], glioma [14] and melanoma [15]. While a mutant form of CDK4 associated with insensitivity towards the inhibitory INK4 proteins is found in familiar melanoma [15], no mutations have been identified so far in CDK6. Alterations for CDK6 include overexpression, which is frequently found in hematologic malignancies [16,17]. The unique functions of CDK6 as a transcriptional regulator in the hematopoietic system, which is not shared by CDK4 may partly explain this pattern.

CDK6 may exert tumor promoting and tumor suppressing functions. On the one hand, the kinase-dependent function of CDK6 in the cell cycle machinery is exploited by rapidly proliferating tumor cells to rush through the cell cycle. In addition, the function of CDK6 as transcriptional regulator interferes with tumor progression at multiple levels. As transcriptional regulator, CDK6 together with the activator protein 1 (AP-1) member c-JUN, controls vascular endothelial growth factor A (*VEGF-A*) expression to regulate angiogenesis [18]. AP-1 is a dimeric complex composed of members of the JUN, FOS, ATF and MAF protein families. Different AP-1 dimers bind distinct DNA sites to regulate transcription of their target genes [19]. The proto-oncogene c-Jun not only drives angiogenesis together with CDK6 but may also regulate the expression of CDK6 itself. C-Jun binds to the *CDK6* promoter region and thereby prevents promoter methylation and thus silencing to maintain high *CDK6* expression levels. The strong interplay of CDK6 and c-Jun leads to the suggestion that CDK6 might also interact with other AP-1 members to fulfill specific functions. This relationship needs to be analyzed in great detail for different disease entities to understand and develop novel treatment options.

At the flip side of the coin, CDK6 suppresses tumorigenesis by forming complexes with STAT3 and cyclin D to induce expression of the tumor suppressor p16^INK4a^ [18,20]. Thereby, CDK6 induces its own inhibitor p16^INK4a^, which is viewed as a feedback loop and internal control mechanism protecting the cell from high CDK6 levels and uncontrolled cell proliferation. Thus, the presence of p16^INK4a^ determines whether CDK6 acts as a tumor-suppressor. In the absence of p16^INK4A^ CDK6 turns into a proto-oncogene [18,20]. In addition, the balanced expression of *CDK4* and *CDK6* is crucial to allow CDK6 to act as transcriptional regulator as has been shown in melanoma cells [21]. Only the presence of CDK4, which takes over cell cycle regulation, frees CDK6 to act as a transcriptional regulator [21].

A high expression of Cyclin D1 and CDK4 is frequently found in breast cancer and deregulation of this pathway contributes to uncontrolled proliferation in hormone receptor-positive breast cancer [22,23,24,25]. Estradiol allows for the rapid progression through the G1 phase of the cell cycle by mediating activation of G1 CDKs, enhancing expression of cyclin D1 and inactivation of the CDK inhibitor p27^KIP^ [26]. In prostate cancer, androgen receptor-dependent transcription is stimulated by direct binding of CDK6 [27]. Overexpression of *CDK6* thereby contributes to prostate cancer progression.

The frequent overexpression of *CDK6* in leukemia and lymphoma has put a focus on CDK6 dependent functions in the hematopoietic system. CDK6 binds RUNX1, a master regulator of hematopoietic differentiation and blocks RUNX1 binding to DNA and thereby myeloid differentiation [28]. Interference with myeloid differentiation was also observed in MLL/AF9-driven myeloid leukemia, where CDK6 inhibition triggered differentiation [29].

## 3. The Role of MAPKs in Cancer

MAPKs are activated by various stimuli and are involved in growth control; it is thus not surprising that dysregulation is linked to initiation and maintenance of numerous cancer types. The Ras/Raf/MEK/ERK signaling cascade is predominantly activated by growth factors and promotes cell proliferation and survival [30,31]. As such, it represents one of the most frequently mutated pathways in cancer [30]. Mutations occur in different components of the pathway with *RAS* and *RAF* being mutational hotspots and considered oncogenic drivers. They are most commonly mutated in lung, colon, pancreatic cancer and melanoma [30,32]. Mutations keep the Ras family of GTPases in an active GTP bound state [32] and predominantly affect *KRAS* [33]. Mutations in *BRAF* are the most common *RAF* mutations with one specific point mutation (*BRAF^V600E^*) being present in more than 90% of cases [33,34]. Mutations of *MEK* and *ERK* are less frequent, but are associated with a poor prognosis in many cancer types and do not co-occur with *RAS* or *RAF* mutations [33].

Aberrant ERK signaling is also described in hematopoietic malignancies and promotes cell growth and survival [35]. A large subset of primary AML shows constitutively activated ERK signaling indicative for a crucial role in leukemogenesis [36]. In chronic myeloid leukemia (CML), the oncogenic fusion kinase BCR-ABL activates MAPK pathways that are suggested to promote leukemogenesis [32]. BCR-ABL inhibition is synergistic with inhibition of the ERK pathway and induces apoptosis in BCR-ABL positive cells [37].

**Figure 2:**
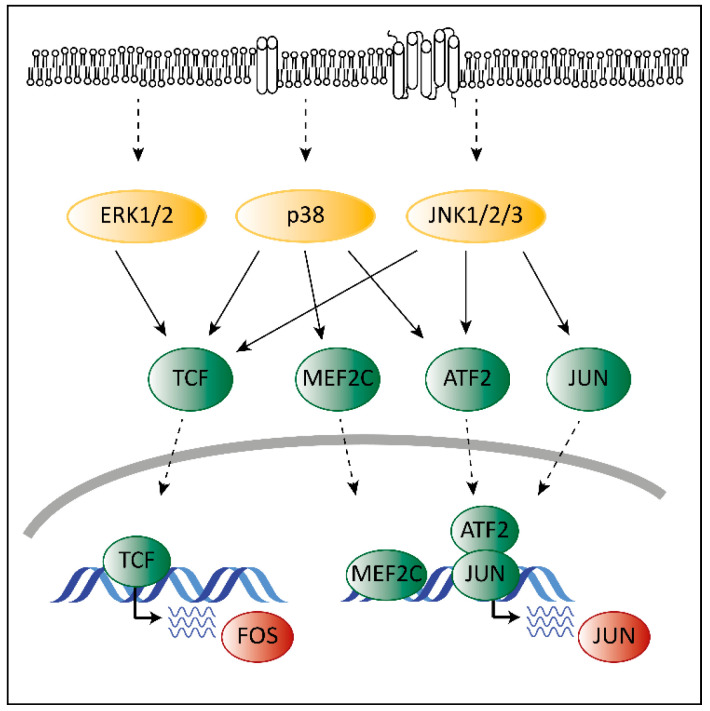
The interplay of CDK4/6 and the MAPK pathways in the context of AP-1. Adapted from [19,41]. Activating signals pass through the MAPK cascade until they reach MAPKs (indicated by the dashed arrows at the top). MAPKs activate ternary complex factors (TCF), MEF2C, ATF2 and JUN (indicated by the arrows) that translocate into the nucleus (indicated by the lower dashed arrows) and induce expression of *FOS* and *JUN*.

Deregulated ERK signaling activates transcription factors that contribute to transformation. Transcription and activation of proto-oncogenic members of the AP-1 family depends on MAPK signaling. Mutations in the MAPK pathway affect AP-1 functions, which contributes to transformation. ERK, p38 and JNK regulate the expression of different AP-1 members via ternary complex factors (TCFs; Figure 2). AP-1 members are also subject to phosphorylation by the MAPK pathway as has been shown for the c-FOS protein [38]. Another example is the activating transcription factor 2 (ATF2), which is a direct target of p38 [39]. Phosphorylated ATF2 forms heterodimers with members of the Jun family to associate with AP-1 binding sites, further enhancing expression of c-Jun [40].

Members of the AP-1 family interact with CDK6 on chromatin to regulate transcription [18]. AP-1 transcription factors might thereby represent the link between the MAPK pathway and CDK6 to enable the crosstalk between these programs contributing to transformation and tumor maintenance.

## 4. The Unique Dual Role of p38

p38 MAPK has been classified as a tumor-suppressor based on its proapoptotic and growth inhibitory functions but may act as a tumor promoter under certain conditions. This dual role of p38 most likely accounts for the fact that p38 is rarely mutated or inactivated in cancer [42]. During early phases of tumorigenesis, p38 suppresses tumor formation by inducing apoptosis in response to stress including carcinogens [42,43], reactive oxygen species (ROS) [44,45,46] or oncogene expression [47,48]. At later time points p38 may switch gear and promote tumor formation by driving gene expression programs characteristic for transformed cells. The dual function of p38 is best exemplified in breast cancer where active p38 suppresses the initiation of mammary tumorigenesis [49] but later on is associated with shorter survival and poor prognosis [50]. To date, what triggers the switch from a tumor suppressor to tumor promoter remains elusive and needs further investigation.

During transformation, cancer cells are highly sensitive to signals activating stress activated protein kinases (SAPKs, JNK and p38). Low signal intensities are required for stimulation compared to non-transformed cells and trigger apoptosis building a barrier against malignant transformation [51]. In transformed cells, cytostatic drugs including cisplatin or etoposide specifically activate SAPKs and induce apoptosis due to enhanced ROS levels [51,52]. In colon cancer cells, cisplatin induces a pronounced p38 activation provoking ROS accumulation and p53 activation [53]. Similar effects were observed in the CML cell line K562 where p38 is required for apoptosis induction upon imatinib treatment [54]. In K562 cells, p38 inhibition promotes myeloid cell differentiation and apoptosis via enhanced JNK activation upon arsenic trioxide. This shows the high complexity of MAPK signaling cross-talks [55]. Similarly, p38 inhibition enhances the chemotherapeutic effects of taxanes and irinotecan in breast and colon cancer models, respectively [56,57]. P38 phosphorylation was even considered a biomarker indicative of irinotecan-resistance in colon cancer [57]. We are only at the beginning of understanding how p38 shapes tumor formation and therapeutic responses.

## 5. CDK6 as a Target in Cancer Therapy

**Figure 3:**
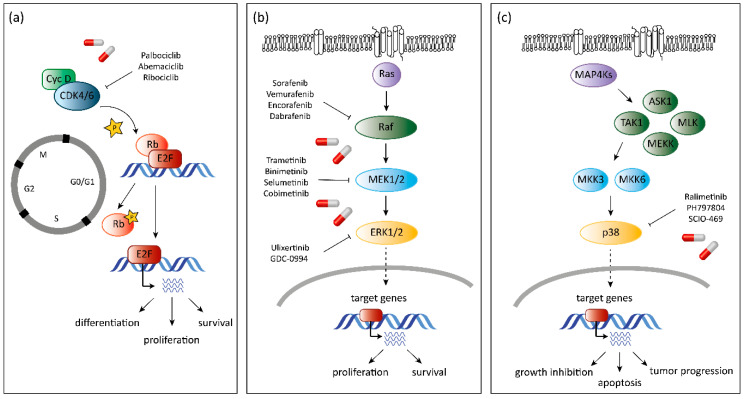
Overview of CDK4/6 and MAPK inhibitors. (**a**) FDA approved small-molecule inhibitors palbociclib, abemaciclib and ribociclib bind to CDK4/6 and prevent phosphorylation of Rb (indicated by the star symbol), which can result in cell cycle arrest in the G1 phase, block of differentiation and survival. (**b**) Inhibitors of the ERK pathway blocking the prosurvival and proliferative effects of ERK signaling. (**c**) Inhibitors of p38 are controversial and can have opposing effects as p38 is a tumor suppressor but can also have tumor promoting effects at later stages of cancer development. “T-lines” indicate inhibition, arrows indicate activation and dashed arrows indicate translocation into the nucleus. The arrows at the bottom of each panel show major outcomes of the signaling networks.

Cell proliferation is tightly controlled and alterations in the regulatory network driving the cell cycle machinery frequently promote tumorigenesis. Aberrant activation of CDKs is a common event in cancer and CDKs represent obvious drug targets. Apart from non-selective agents that block CDKs, small molecular weight inhibitors with dual specificity for the close homologues CDK4 and CDK6 have been FDA approved for the treatment of breast cancer [25,58,59]. Selective targeting of CDK4/6 by the ATP-competitive inhibitors palbociclib [60], abemaciclib [61] and ribociclib [62] induces cell cycle arrest via prevention of Rb phosphorylation (Figure 3a). Besides the obvious effects on cell cycle arrest, the effects of CDK4/6 inhibition go well beyond and include induction of a senescence-like state and an improved tumor surveillance [63]. Cells harboring alterations or mutations in genes promoting the activation of D-type cyclins are particularly sensitive towards CDK4/6 inhibition [64]. Preclinical studies identified luminal estrogen receptor (ER)-positive cell lines as highly sensitive towards the growth inhibiting effect of CDK4/6 inhibitor palbociclib and verified synergistic effects upon combination with the ER antagonist tamoxifen [65]. The FDA approval of palbociclib for treatment of ER-positive, v-erb-b2 avian erythroblastic leukemia viral oncogene homolog 2 (ErbB2)-negative breast cancer patients was a major breakthrough [66,67,68]. The basis was laid in the phase II PALOMA-1/TRIO-18 clinical study in patients with previously untreated, advanced-stage, ER-positive, ErbB2-negative breast cancer [66]. Patients were randomly assigned for treatment with either the aromatase inhibitor letrozole alone or in combination with palbociclib. Strikingly, the addition of palbociclib to endocrine therapy almost doubled the progression-free survival with the principal toxicity being neutropenia. The follow-up phase III PALOMA-2 study confirmed and expanded the efficacy and safety data for the same treatment [69]. In the phase III PALOMA-3 study, patients with advanced-stage, hormone receptor positive, ErbB2-negative breast cancer that had progressed after endocrine therapy were assigned for treatment with either the endocrine therapeutic agent fulvestrant alone or in combination with palbociclib [68]. The positive results of the studies led to FDA approval of palbociclib in combination with letrozole and fulvestrant as initial or second-line treatments, respectively.

The safety and antitumor efficacy of ribociclib in breast cancer patients was assessed in the MONALEESA clinical program, which led to the FDA approval of ribociclib in this context [70]. This study series enrolled patients with hormone receptor positive, ErbB2-negative advanced breast cancer. MONALEESA-2 and 3 trials showed improved progression-free survival of patients treated with ribociclib in combination with letrozole or fulvestrant as first-line therapy or after chemotherapy, respectively [71,72].

Abemaciclib has demonstrated antitumor efficacy and an improved safety profile in a phase I clinical trial in a variety of different tumors even as a single agent [73]. The most promising results were seen in breast cancer and non-small cell lung cancer (NSCLC) patients and prompted further studies. In the phase III JUNIPER study, previously chemotherapy treated patients with advanced stage NSCLC patients with *KRAS* mutations showed improved response rates and progression-free survival, which supports further investigations [74]. The MONARCH study series enrolled patients with hormone receptor positive, ErbB2-negative advanced breast cancer and led to FDA approval of abemaciclib as initial endocrine-based therapy in combination with an aromatase inhibitor [75]. Further, abemaciclib was approved as second-line treatment either in combination with fulvestrant or as monotherapy [76,77].

Even though the three inhibitors all have CDK4 and CDK6 as primary kinase targets, novel studies describe significant differences in the transcriptional, proteomic and phenotypic changes induced by palbociclib, ribociclib and abemaciclib [78]. While palbociclib and ribociclib have similar selectivity and efficacy profiles, abemacilib has greater selectivity for CDK4 than CDK6 [61] and generally shows weaker target specificity. Abemaciclib also inhibits CDK1 and CDK2 and thus resembles more a pan-CDK inhibitor [78,79]. This might be an explanation for abemaciclib being the only CDK4/6 inhibitor approved as monotherapy for ER-positive, ErbB2-negative advanced or metastatic breast cancer. Additionally, the toxicity profiles of the three inhibitors differ. The dose-limiting toxicity for palbociclib and ribociclib was determined by neutropenia as a primary adverse event, which led to a treatment schedule that includes a 7 day treatment pause after 3 weeks of inhibitor administration [80]. Abemaciclib can be administered on a continuous schedule as it caused less neutropenia [73].

Apart from the three CDK4/6 inhibitors that have already entered the clinics, there are a number of novel inhibitors developed that have shown impressive antitumor activity in preclinical studies (Table 1). A novel modality of inhibition is explored by MMD37K, the first ATP non-competitive CDK4/6 inhibitor. Its structure is derived from the endogenous CDK4/6 inhibitor p16^INK4a^ coupled to a cell penetrating sequence. MMD37K has shown a promising cytotoxic, cytostatic and antitumor effect in vitro and in vivo [81]. Another new CDK4/6 inhibitor, trilaciclib, prevents chemotherapy-induced myelosuppression. It arrests hematopoietic stem and progenitor cells in the bone marrow in the G1 phase during chemotherapy treatment, protecting them from chemotherapy-induced damage [82,83]. Trilaciclib is in this context beneficial over the other CDK4/6 inhibitors as it can be administered intravenously and has a shorter half-life that matches the duration of the chemotherapy treatment [82]. Several clinical trials with trilaciclib are currently ongoing in patients with hormone receptor negative breast cancer and small cell lung cancer (NCT03041311 and NCT02978716). First phase I/II clinical trials already showed a better chemotherapy tolerability of patients treated with trilaciclib [84,85]. Additionally, SHR6390, a novel CDK4/6 inhibitor has recently entered clinical studies (Table 1). SHR6390 has shown potent antitumor activity in different tumor models and favorable pharmacokinetics and pharmacodynamics in preclinical studies [86,87].

Overall, a deeper understanding of biomarkers of response and resistance and accurate definitions of pharmacokinetics and pharmacodynamics for all inhibitors are needed. Due to unique binding strategies and activities of the CDK4/6 inhibitors, we would expect different inhibitions of distinct CDK6 complexes and therefore of specific CDK6 functions. Only an exact analysis and understanding of the overlapping and unique consequences of treatments with different inhibitors will enable the optimization of treatment schedules and expansion of administration of CDK4/6 inhibitors to various types of cancers.

The synergistic effects of endocrine therapy and CDK4/6 inhibitors are considered to result from blocking CDK4/6 while in parallel reducing their binding partner, the D type cyclins. Endocrine therapy reduces ER signaling, which dampens D type cyclin expression [88,89]. Inhibition of CDK4/6 by small molecular inhibitors further reduces Rb-phosphorylation to block proliferation [63]. Nevertheless, the effect of CDK4/6 inhibition extends to breast cancer patients with acquired resistance to endocrine therapy. Usually, ER signaling can be inhibited by antiestrogens. However, there is also a hormone-independent transcriptional program induced by ER that activates E2F target genes, which is prevented by CDK4/6 inhibition [90]. This dual effect of CDK4/6 inhibitors on cell cycle control and transcriptional regulation is currently exploited in many clinical studies. Single-arm studies evidenced clinical activity of CDK4/6 inhibitors in a subset of patients suffering from mantle cell lymphoma, liposarcoma, melanoma, non-small cell lung cancer, glioblastoma, neuroblastoma and malignant rhabdoid tumors [73,91,92,93].

As CDK6 is frequently overexpressed in hematologic malignancies [16,17], inhibition of CDK6 appears a promising treatment option and numerous clinical studies are underway. Several preclinical evaluations showed the effects of CDK4/6 inhibitor treatment in different leukemia entities [94]. *FLT3*-mutated human AML cell lines are particularly sensitive towards palbociclib treatment and react with apoptosis induction, which was verified in murine leukemia models [95]. An increased cytotoxicity is induced upon combinatorial treatment with palbociclib and FLT3 inhibitors [96]. The promising potential of the dual inhibition was exploited to develop AMG 925, a dual inhibitor for FLT3 and CDK4/6 that is currently tested [97,98]. In MLL-rearranged AML, CDK6 inhibition results in growth inhibition and myeloid differentiation and palbociclib treatment induces prolonged survival of mice in vivo [29]. Clinical trials in MLL-rearranged leukemia are underway (NCT02310243).

CDK4/6 inhibitors target the kinase activity leaving kinase-independent effects unaffected. This is of relevance as some of the CDK6-mediated transcriptional effects are exerted in a kinase-independent manner. A prominent example is *JAK2^V617F^*-driven myeloproliferative neoplasm (MPN), where CDK4/6 kinase inhibition does not mimic the anticancer effects induced by deletion of *CDK6* [99]. Targeting kinase-dependent and kinase-independent effects of CDK6 may be thus beneficial for MPN patients and patients suffering from other cancer types. Homologue-selective degraders for CDK6 have been developed and were able to significantly reduce leukemia burden in xeno-transplant experiments of Philadelphia chromosome-positive acute lymphoid leukemia with superior effects compared to CDK4/6 kinase inhibition [100]. Their clinical impact remains to be determined. When treating patients with CDK4/6 inhibitors, the fact that CDK6 antagonizes p53 responses under oncogenic stress needs to be taken into consideration. Low levels of CDK6 pressure the cells to mutate p53, which can currently not be entirely excluded to occur also in cancer patients treated with CDK6 inhibitors [101].

When combined with other drugs, some peculiarities of CDK4/6 inhibition must be taken into account. CDK4/6 inhibitor treatment may dampen the effects of cytostatic and cytotoxic drugs [102] as some cytostatic drugs interfere with DNA replication in the S phase, which is prevented upon CDK4/6 inhibition. On the other hand, when applied sequentially CDK4/6 inhibitors have recently been shown to enhance cytotoxicity by preventing recovery of cancer cells as shown in pancreas cancer. CDK4/6 inhibition is considered to interfere with expression of proteins involved in homologous recombination that are required for recovery after drug-induced DNA-damage [103].

## 6. Resistance to CDK4/6 Inhibitors

Despite the success of CDK4/6 inhibitors, there is on the one hand a significant subset of treated patients that has an innate resistance and does not respond. On the other hand, prolonged treatment eventually provokes resistance, which has been attributed to the loss of the Rb tumor-suppressor or cyclin D1, activation of CDK2, cyclin E1 and amplification of *CDK6* [104,105,106]. The identification of biomarkers predicting the responsiveness towards CDK4/6 inhibitors is of great importance [79,107]. Factors that might predict primary resistance were already assessed during early clinical trials of palbociclib. As high expression levels of Cyclin D1 and CDK4 are frequently found in breast cancer and contribute to uncontrolled proliferation, they are potential candidates for biomarkers. Even though preclinical data suggested a correlation between amplification of *CCND1*, the gene encoding cyclin D1 and sensitivity towards palbociclib [65], *CCND1* levels did not qualify as predictive biomarkers in patients [66,108]. Higher levels of activated CDK4 are linked to a better response to palbociclib treatment [109]. In the clinics, patients with high CDK4 levels are prone to endocrine therapy resistance, which can be reduced by CDK4/6 inhibitor treatment [110]. Amplifications of *CDK6* were also found in breast cancer cell lines with acquired abemaciclib resistance and knock-down of *CDK6* was sufficient to restore sensitivity [111]. As innate inhibitor of CDK4/6, also p16^INK4a^ might serve as a biomarker for responsiveness towards CDK4/6 inhibitors. Low levels of p16^INK4a^ lead to enhanced CDK4/6 activity suggesting a higher sensitivity towards CDK4/6 inhibitors. However, expression levels of p16^INK4a^ were not predictive for response to treatment [66,108].

Further downstream of CDK6, Rb regulates the expression of E2F target genes and is thus a possible biomarker that is independent of the CDK4/6 status. High levels of Rb are correlated with higher sensitivity towards CDK4/6 inhibitors in preclinical studies and downregulation of Rb is considered as a mechanism of acquired palbociclib resistance [65,112,113]. In hormone receptor positive breast cancer patients, loss of *RB* is relatively rare [66,110]. These patients, however, benefitted less from CDK4/6 inhibitor treatment than patients with intact *RB* [114].

The CDK2–cyclin E complex is not only downstream of CDK4/6–cyclin D in cell cycle progression but is also able to compensate for CDK4/6 inhibition in phosphorylating Rb [115,116]. Thus, changes in the CDK2–cyclin E complex might be involved in both primary and acquired resistance to CDK4/6 inhibitors. High levels of cyclin E1 were found in hormone receptor positive breast cancer cell lines with acquired resistance to palbociclib [117,118] and the cyclin E1/Rb ratio was proposed as a marker for palbociclib resistance [118]. Additionally, in the clinics, patients with low cyclin E1 levels respond better to palbociclib treatment compared to those with cyclin E1 overexpression [119].

The definition and detailed understanding of biomarkers of treatment response and resistance mechanisms is of great importance and will give rise to new therapeutic options that prevent or overcome resistance. Resensitization of tumor cells with acquired resistance has been shown in breast cancer patients with acquired resistance to endocrine therapy [90].

## 7. MAPK as Target in Cancer Therapy

With the ERK signaling cascade being frequently mutated, efforts to target its components have a long-standing history. Ras has long been considered undruggable, due to the absence of any deep hydrophobic pocket on its surface that could serve as a binding site for a small molecular inhibitor [33,120]. Approaches to inhibit farnesyltransferases that are required to link RAS to the cell membrane by post-translational modification failed [121] as KRAS and NRAS were able to circumvent farnesyltransferase to get post-translationally modified and located to the membrane [30,122]. A new inhibitor targets a recently described pocket on the surface of the *KRAS^G12C^* mutant [123]. Clinical trials in patients with advanced *KRAS^G12C^*-mutant solid tumors are ongoing [124] (NCT03600883).

The kinase Raf offers therapeutic opportunities more readily. Sorafenib was a first-generation Raf inhibitor designed to inhibit Raf-1 [31,125,126] (Figure 3b). Sorafenib also showed activity against B-Raf and other protein kinases including VEGFR, PDGFR and Flt3 [127]. Sorafenib was first approved by the FDA in 2005 for the treatment of renal cell carcinoma [128], which was later extended to the treatment of advanced stage hepatocellular and thyroid carcinoma. The predominant mutation of *BRAF* prompted the development of the BRAF inhibitors vemurafenib [129] and dabrafenib [130], which were FDA approved as mono-therapeutic agents in *BRAF^V600E^*-mutant melanoma. The Raf inhibitor encorafenib [131] has recently entered the clinics for the treatment of metastatic colorectal cancer with *BRAF^V600E^* mutations in combination with the MEK inhibitor binimetinib [132] (Table 2). The combination is designed to block resistance development.

Resistance towards Raf inhibitors evolves months after initiation of therapy and is related to reactivation of the ERK pathway, overexpression of the epidermal growth factor receptor activating Ras or upregulation of the PI3K pathway [12,139,140,141]. Key mechanisms of ERK signaling reactivation include mutations in *RAS* and *MEK* and amplification of *BRAF* [142,143]. Simultaneous blocking of Raf and MEK prevents reactivation of ERK signaling and delays resistance development [144]. Several combinations to block RAF and MEK have been approved already for the treatment of cancers harboring *BRAF* mutations (Table 2).

Inhibition of ERK is used to prevent the development of resistance caused by reactivation of the ERK pathway upon Ras or MEK blockade. Preclinical studies in *KRAS*-mutant murine tumor models showed synergistic effects upon combined inhibition of MEK and ERK [145]. This prompted clinical trials with the ERK inhibitor GDC-0994 for advanced or metastatic solid tumor patients (Table 3). In *BRAF*^V600E^-mutant melanoma xenograft models the ERK inhibitor ulixertinib (BVD-523) showed impressive synergistic growth-inhibitory effects upon combination with BRAF inhibitors. The effects extended to in vivo models with acquired resistance to BRAF and MEK mono- or combinational therapies [146]. Ulixertinib is therefore currently explored in clinical trials [147] (Table 3). Further ERK inhibitors are currently tested in clinical trials alone or in combination with other agents [148] (Table 3).

The tumor suppressive and tumor promoting role of p38 MAPK renders p38 a complex therapeutic target [42] (Figure 3c). Numerous preclinical studies explored consequences of p38 inhibition. Lung cancer appears to be sensitive to p38 inhibition. *KRAS*-mutant NSCLC cells react with synergistic antitumor effects to the MEK inhibitor selumetinib and the p38 inhibitor ralimetinib [149]. In line, epithelial p38 was assigned a crucial role in the progression of *KRAS^G12V^*-driven lung cancer and p38 has been implicated in transformation and proliferation of lung cancer cells in in vivo models. High levels of p38 correlate with poor survival in lung adenocarcinoma patients [150]. Of note, studies combining p38 inhibition in conjunction with chemotherapeutic agents such as cisplatin, irinotecan or arsenic trioxide showed opposing results and led to the acceleration of tumor growth upon p38 inhibition [53,54,55,57,151,152,153]. No p38 inhibitor has been approved for clinical use but clinical trials are ongoing in advanced cancer patients with the p38 inhibitor ralimetinib (Table 3). In addition, the p38 inhibitor SCIO-469 is currently tested in phase II studies in patients with multiple myeloma or myelodysplastic syndrome (MDS; Table 3).

## 8. Combinatorial Treatment with CDK4/6 and MAPK Inhibitors

The successful combination of endocrine therapy and CDK4/6 inhibition in breast cancer is considered to be the consequence of ER-signaling mediated reduction of cyclin D1 expression and blocking CDK4/6 kinase function [88,89]. Other factors that influence the expression of cyclin D1 are thus debated as potential therapeutic targets to be combined with CDK4/6 inhibitors. RAS/RAF/MEK/ERK signaling that regulates cyclin D1 expression is one opportunity [154]. The complex functions of the ERK pathway on tumor formation and the additional transcriptional role of CDK6 make it likely that additional effects contribute to the pronounced tumor inhibitory effects.

This is also evident in preclinical studies that revealed synergistic effects upon combination of RAF inhibitors with the CDK4/6 inhibitor abemaciclib. This combination led to tumor regression in xenograft models with *KRAS*, *NRAS* or *BRAF* mutations [155]. Although the synergistic effects were discussed to result from pronounced inhibition of Rb phosphorylation [155] also *BRAF*-mutant melanoma xenograft models with acquired resistance towards the BRAF inhibitor vemurafenib led to regression of tumor growth upon combination. Abemaciclib treatment induces apoptosis in BRAF inhibitor resistant cell lines, whereas it induces cell cycle arrest in non-resistant cells [156].

**Figure 4:**
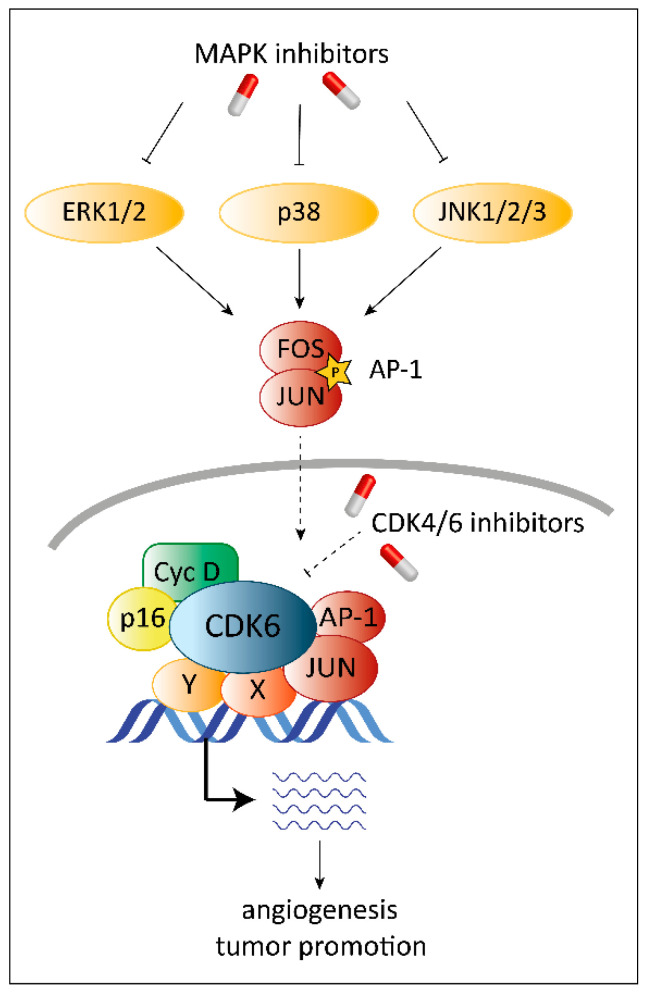
Regulation of transcription mediated by CDK6 and AP-1. CDK6 is not only a cell cycle kinase but also has transcriptional functions. Together with cofactors like JUN (indicated by the star symbol) and potentially also other AP-1 factors it regulates transcription of target genes like *VEGF-A* [18]. Inhibition of MAPK (indicated by the “T-lines”) blocks activation of AP-1 (indicated by the arrows) and its translocation to the nucleus (indicated by the dashed arrow) and thus might interfere with tumor promoting transcriptional complexes containing CDK6. Blocking of CDK6 with small-molecule inhibitors only inhibits kinase-dependent functions. The transcriptional roles of CDK6 can be dependent or independent of its kinase activity. Thus, treatment with CDK4/6 inhibitors only partially blocks the transcriptional functions of CDK6 (indicated by the dashed “T-line”).

The functions of CDK6 go well beyond its role as a cell cycle kinase. Any explanation for potential synergistic effects of MAPK and CDK4/6 inhibitors must consider the transcriptional activity of CDK6. On the DNA level, CDK6 interacts with the AP-1 member c-Jun to induce expression of *VEGF-A* promoting angiogenesis [18]. It is tempting to speculate that c-Jun might not be the only AP-1 transcription factor cooperating with CDK6 in regulation of oncogenic transcriptional programs (Figure 4). Given the fact that MAPKs not only regulate the expression of AP-1 transcription factors but also activate them, inhibition might also interfere with their activity to regulate transcription in conjunction with CDK6. When combined with MAPK-inhibitors, CDK4/6 inhibitors may not only block the kinase activity of CDK6 but may also interfere with kinase-independent transcriptional functions of CDK6 indirectly blocked by MAPK inhibitors via interfering with AP-1 activity.

An additional concept for the synergistic effects of CDK4/6 and MAPK inhibition has been described in melanoma where CDK6 mediates resistance towards BRAF inhibitor treatment. Resistance towards BRAF inhibitors is restricted to enhanced *CDK6* expression and not shared by other CDKs. BRAF inhibitor resistant melanoma cells display elevated CDK6 levels paralleled by increased expression and chromatin binding of the CDK6 partner c-Jun [157]. C-Jun itself mediates BRAF inhibitor resistance and promotes invasion and cell survival during drug adaptation [158]. The interaction of c-Jun with CDK6 may be the molecular link explaining BRAF inhibitor resistance in the presence of high CDK6 levels. The key role of CDK6 is also evident as combined treatment of the BRAF inhibitor resistant melanoma cell lines with palbociclib and vemurafenib resensitized the cells [157].

These findings were replicated in murine xenograft models of mucosal melanoma where combined inhibition of MEK and CDK4/6 in cells harboring oncogenic BRAF fusions significantly reduced tumor growth [159]. Similarly, combinational therapy with trametinib and palbociclib significantly reduced tumor size in *NRAS*-mutant melanoma transplantation models [160]. Accordingly, in a phase I clinical study, the combination of ribociclib and binimetinib in *NRAS*-mutant melanoma showed encouraging clinical activity [161]. Clinical trials are currently probing treatment with BRAF and MEK inhibitors in combination with ribociclib in *BRAF*-mutant melanoma and other solid tumors with *BRAF^V600^* mutations (Table 4). It is currently unclear how *NRAS* and *MEK* mutations interfere with RB-mediated cell cycle checkpoint control [160]. MEK inhibitors can only incompletely block cell cycle progression compared to RAS inhibition, therefore a combination with CDK4/6 inhibitors may be of advantage. This treatment combination could at least partially compensate for the lack of RAS inhibitors.

Synergistic effects were also observed in xenograft models of *KRAS*-mutant colorectal cancer upon treatment with a combination of trametinib and palbociclib where a pronounced tumor regression in vivo was observed [162]. This prompted a phase II clinical trial in *KRAS-* or *NRAS*-mutant colorectal cancer patients testing binimetinib and palbociclib in combination (Table 4).

The *KRAS* oncogene is one of the most frequently mutated genes in NSCLC and renders treatment difficult. NSCLC frequently harbors mutations in cyclin D/CDK4/6/Rb signaling pathways. A synergistic growth inhibition was obtained in a *RAS*-driven NSCLC xenograft model upon treatment with the MEK inhibitor selumetinib in combination with palbociclib [163]. Palbociclib treatment induced downregulation of survivin, an effect that depends on the mutational status of *CDKN2A*, encoding the CDK inhibitor p16^INK4a^. Cells with mutated *CDKN2A* hyperproliferate independently of high CDK4/6 levels. Mutations in p16^INK4a^ might influence the composition of transcriptional complexes involving CDK6. Palbociclib treatment in *CDKN2A*-deleted tumors mimics p16^INK4A^ inhibition as palbociclib targets kinase monomers, which is not competed by *CDKN2A* in mutant cells [164,165]. Currently, a phase I/II clinical study in advanced *KRAS*-driven NSCLC patients is investigating the combinational treatment with palbociclib and binimetinib (Table 4).

In neuroblastoma, activating mutations of the MAPK pathway are a common feature upon relapse [166]. Sensitivity towards treatment with MEK inhibitors and CDK4/6 inhibition seems to have an inverse correlation. Combinational treatment of binimetinib and ribociclib inhibited tumor growth in a synergistic manner in neuroblastoma in xenograft models [167]. These results provoked a clinical trial in relapsed neuroblastoma patients harboring activating MAPK mutations treated with trametinib and ribociclib (Table 4).

CDK4/6 inhibition is well established and considered a major success for ER-positive, ErbB2-negative patients. For the treatment of ErbB2-positive breast cancers, a new drug is under development. Pyrotinib acts as a pan-HER kinase inhibitor and concomitantly suppresses RAS/RAF/MEK/ERK signaling. First results in a phase I clinical trial are promising [168,169]. Moreover, a combination of pyrotinib in combination with palbociclib, showed synergistic antitumor activity in a preclinical xenograft model [170].

Besides CDK4/6, cyclin D1 may represent a valuable target for cancer therapy. *CCND1* itself is considered a proto-oncogene and is frequently amplified and overexpressed in various cancers [171]. Cyclin D1 knockout mice are resistant to breast cancer initiation despite expression of oncogenes like *ErbB2* or *Ras* [172]. Pharmacologically, D-type cyclins are difficult to target as they lack enzymatic activity. Thus, cyclin D1 may be indirectly targeted by compounds that lead to reduced expression of *CCND1* or inhibit its primary binding partner CDK4/6 [173].

In summary, the combinatorial inhibition of the MAPK pathway and CDK4/6 activation shows promising results in preclinical studies and in first clinical studies. The beneficial effects are attributed to cell cycle inhibition and an altered transcriptional regulation of CDK6 and AP-1. Fighting cancer at two fronts by interfering with two distinct regulatory networks should benefit patients and help to prevent resistance development.

## 9. Future Perspectives

The MAPK pathway and the cell cycle machinery are among the most frequently mutated cellular networks in cancer, which are now successfully targeted by small-molecular weight inhibitors. Novel therapeutic opportunities may evolve from a better understanding of the tumor promoting role of the p38 MAPK cascade. Another area for new developments is combinational therapy to prevent and overcome drug resistance. Resistance towards CDK4/6 inhibitors is associated with MAPK activation and subsequent sensitization towards MEK inhibition. Thus, combining CDK4/6 inhibitors with MAPK inhibition seems a particular promising approach [174]. It will be important to identify biomarkers to define who benefits from such combinational treatment. The mutational status of *RB* and *CDKN2A* represents starting points. To understand the interaction of CDK6-AP1 complexes and their regulation of oncogenic gene expression programs opens another field of novel opportunities, which will be facilitated by the development of degraders specific for CDK6.

## Figures and Tables

**Table 1:** Overview of novel CDK4/6 inhibitors in clinical trials.

Clinicaltrials.gov Identifier.	Phase	Treatment	Drug Target	Target Disease	Primary Results
NCT03041311	II	trilaciclib plus etoposide/carboplatin/atezolizumab	CDK4/6 plus chemotherapy/immune therapy	small cell lung cancer	N/A
NCT02514447	Ib/IIa	trilaciclib plus topotecan	CDK4/6 plus chemotherapy	small cell lung cancer	better tolerability of chemotherapy
NCT02499770	Ib/IIa	trilaciclib plus etoposide/carboplatin	CDK4/6 plus chemotherapy	small cell lung cancer	better tolerability of chemotherapy
NCT02978716	II	trilaciclib plus gemcitabine plus carboplatin	CDK4/6 plus chemotherapy	hormone receptor negative breast cancer	N/A
NCT03480256	I	SHR6390 plus pyrotinib	CDK4/6 plus ErbB	ErbB2 positive gastric cancer	partial response
NCT04095390	II	SHR6390 plus pyrotinib plus letrozole/capecitabine	CDK4/6 plus ErbB plus endocrine therapy/chemotherapy	ErbB2 positive breast cancer	N/A
NCT03772353	Ib/II	SHR6390 plus pyrotinib plus letrozole	CDK4/6 plus ErbB plus endocrine therapy	hormone receptor positive, ErbB2 positive breast cancer	N/A
NCT03481998	Ib/II	SHR6390 plus letrozole/anastrozole/fulvestrant	CDK4/6 plus endocrine therapy	hormone receptor positive, ErbB2 negative breast cancer	N/A
NCT03966898	III	SHR6390 plus letrozole/anastrozole	CDK4/6 plus endocrine therapy	hormone receptor positive, ErbB2 negative breast cancer	N/A

N/A = not available.

**Table 2:** Overview on FDA-approved combined or single-agent MEK inhibitor treatments.

Raf Inhibitor	MEK Inhibitor	Target Disease	References
dabrafenib	trametinib	melanoma, thyroid cancer, non-small cell lung cancer	[133,134,135]
vemurafenib	cobimetinib	melanoma	[136]
encorafenib	binimetinib	melanoma, metastatic colorectal cancer with *BRAF^V600E^* mutations	[132,137]
	trametinib	advanced melanoma with *BRAF^V600E^* and *BRAF^V600K^* mutations	[138]

**Table 3:** Overview of clinical trials with novel MAPK inhibitors.

Clinicaltrials.gov Identifier	Phase	Treatment	Drug Target	Target Disease	Primary Results
NCT03600883	I/II	AMG 510	KRAS G12C	advanced solid tumors with KRAS p.G12C mutation	N/A
NCT01875705	I	GDC-0994	ERK	advanced or metastatic solid tumors	partial response
NCT02457793	I	GDC-0994 plus cobimetinib	ERK plus MEK	advanced or metastatic solid tumors	overlapping adverse events
NCT01781429	I	BVD-523 (ulixertinib)	ERK	advanced solid tumors	partial response
NCT02296242	I/II	BVD-523 (ulixertinib)	ERK	AML, MDS	N/A
NCT04198818	I/II	HH2710	ERK	advanced solid tumors	N/A
NCT04081259	I	LY3214996	ERK	AML	N/A
NCT01393990	I	LY2228820 (ralimetinib) monotherapy or LY2228820 plus midazolam/tamoxifen	p38/sedative/endocrine therapy	advanced tumors	N/A
NCT01663857	Ib/II	LY2228820 (ralimetinib) plus gemcitabine plus carboplatin	p38 plus chemotherapy	recurrent platinum-sensitive ovarian cancer	improvement of progression free survival
NCT02860780	I	LY2228820 (ralimetinib) plus prexasertib	p38 plus CHK1	advanced or metastatic tumors	N/A
NCT00095680	II	SCIO-469 monotherapy or SCIO-469 plus bortezomib	p38/proteasome	multiple myeloma	N/A
NCT00113893	II	SCIO-469	p38	MDS, hematologic diseases	N/A

N/A = not available; AML = acute myeloid leukemia; MDS = myelodysplastic syndrome.

**Table 4:** Overview of ongoing clinical trials with combined CDK4/6 and MAPK inhibitor treatment.

Clinicaltrials.gov Identifier	Phase	Treatment	Drug Target	Target Disease	Primary Results
NCT02159066	II	LGX818 (encorafenib) plus binimetinib plus ribociclib	BRAF plus MEK1/2 plus CDK4/6	*BRAF*-mutated melanoma	N/A
NCT01543698	Ib/II	LGX818 (encorafenib) plus binimetinib plus ribociclib	BRAF plus MEK1/2 plus CDK4/6	tumors with *BRAF^V600^* mutations	N/A
NCT03981614	II	binimetinib plus palbociclib	MEK1/2 plus CDK4/6	advanced stage colorectal carcinoma with *NRAS* or *KRAS* mutations	N/A
NCT03170206	I/II	binimetinib plus palbociclib	MEK1/2 plus CDK4/6	advanced stage NSCLC with *KRAS* mutations	N/A
NCT02780128	I	ceritinib plus trametinib plus ribociclib	ALK plus MEK1/2 plus CDK4/6	relapsed neuroblastoma with *ALK* or *MAPK* mutations	N/A
NCT03454035	I	BVD-523 (ulixertinib) plus palbociclib	ERK plus CDK4/6	pancreatic cancer and other solid tumors	N/A
NCT02065063	I/II	palbociclib plus trametinib	CDK4/6 plus MEK1/2	solid tumors	N/A
NCT03132454	I	palbociclib plus sorafenib/decitabine/dexamethasone	CDK4/6 plus Raf-1/chemotherapy	recurrent or refractory leukemia	N/A
NCT01781572	Ib/II	ribociclib plus binimetinib	CDK4/6 plus MEK1/2	advanced *NRAS*-mutant melanoma	partial response

N/A = not available; NSCLC = non-small cell lung cancer; ALK = anaplastic lymphoma kinase.
